# Muscle and Tendon Contributions to Reduced Rate of Torque Development in Healthy Older Males

**DOI:** 10.1093/gerona/glx149

**Published:** 2017-08-02

**Authors:** Jonathan I Quinlan, Constantinos N Maganaris, Martino V Franchi, Kenneth Smith, Philip J Atherton, Nathaniel J Szewczyk, Paul L Greenhaff, Bethan E Phillips, James I Blackwell, Catherine Boereboom, John P Williams, John Lund, Marco V Narici

**Affiliations:** 1 Division of Medical Sciences and Graduate Entry Medicine, University of Nottingham, MRC-ARUK Centre for Musculoskeletal Ageing Research, Royal Derby Hospital, UK; 2 Faculty of Sciences, School of Sports and Exercise science, Liverpool John Moore’s Univeristy, UK

**Keywords:** Ageing, Muscle-tendon unit, Tendon Stiffness

## Abstract

**Background:**

The ability to rapidly generate and transfer muscle force is essential for effective corrective movements in order to prevent a fall. The aim of this study was to establish the muscle and tendon contributions to differences in rate of torque development (RTD) between younger (YM) and older males (OM).

**Method:**

Twenty-eight young males (23.9 years ± 1.1) and 22 old males (68.5 years ± 0.5) were recruited for assessment of Quadriceps Anatomical CSA (ACSA), maximal voluntary contraction (MVC), rate of torque development (RTD), and tendon biomechanical properties. Activation capacity (AC), maximal muscle twitch df/dt) and time to peak EMG amplitude (TTPE) were also assessed.

**Results:**

Absolute RTD (aRTD) was lower in OM (577.5 ± 34.6 Nm/s vs 881.7 ± 45.6 Nm/s, *p* < .0001). RTD remained lower in OM following normalization (nRTD) for muscle ACSA (9.93 ± 0.7 Nm/s/cm^2^ vs 11.9 ± 0.6 Nm/s/cm^2^, *p* < .05). Maximal muscle twitch df/dt (1,086 Nm∙s^−1^ vs 2,209 Nm∙s^−1^, *p* < .0001), TTPE (109.2 ± 8.6ms vs 154.6 ± 16.6 ms, *p* < .05), and AC (75.8 ± 1.5% vs 80.1 ± 0.9%, *p* < .01) were all affected in OM. Tendon stiffness was found to be lower in OM (1,222 ± 78.4 N/mm vs 1,771 ± 154.1 N/mm, *p* < .004). nRTD was significantly correlated with tendon stiffness (*R*^2^ = .15).

**Conclusion:**

These observations provide evidence that in absolute terms, a lower RTD in the elderly adults is caused by slower muscle contraction speeds, slower TTPE, reduced ACSA, reduced MVC, and a decrease in tendon stiffness. Once the RTD is normalized to quadriceps ACSA, only MVC and tendon stiffness remain influential. This strongly reinforces the importance of both muscle and tendon characteristics when considering RTD.

Loss of muscle mass and strength (1,2) together with an increased incidence of falls (3) are common outcomes of an ageing neuromuscular system.

While absolute force production is essential for daily activities such as rising from a chair or bath, carrying groceries, or stair negotiation, the ability to generate force rapidly is equally important. In order to counteract a trip and hence prevent a fall, force production must occur extremely rapidly (<200 ms) (4). Therefore, not only maximal torque production but also a rapid rate of torque development (RTD) have been identified as key performance characteristics in elderly individuals (5); significantly, RTD has been shown to decrease in ageing (6–9).

It is possible to assess an individual’s ability to produce an explosive and forceful contraction in vivo, by measuring contractile RTD (10). RTD is defined as the slope of the torque-time curve obtained during an isometric contraction and can be assessed over varying time intervals (11). One factor that may influence RTD, first hypothesized by Wilkie (12), is the compliance of the attached tendon, which provides a mechanical link between muscle and joint. As such, a more extensible or compliant tendon, will take a longer period to stretch and thus reduce the RTD. It has previously been shown that RTD is significantly correlated to the stiffness of the m. vastus lateralis tendon-aponeurosis complex (13), further suggesting that connective tissue may play an important role in explosive strength development. Thus, any changes to the tendon tissue in response to ageing could potentially alter the RTD. Unfortunately, conclusive evidence for changes in tendon tissue with ageing remains elusive. Measurements of tendon biomechanical properties, including tendon stiffness and Young’s modulus have long been acquired in vivo via real-time ultrasonography (14,15) and provide important information on the biomechanical integrity of the tendon. Only a few studies have applied this technique to investigate the effect of ageing; some studies reported that tendon stiffness does not alter during the ageing process (16,17), while others demonstrated that tendon stiffness decreases with age (18). The latter study investigated the gastrocnemius tendon while the former studies were performed on the patellar tendon, pointing to possible tendon-specific differences. Unfortunately, it is difficult to compare tendon tissues from differing anatomical locations as both their properties and consequential adaptations are known to vary, presumably to fulfill its specific requirement (19). It is therefore plausible, that some tendinous tissue may age differently, generating these contradictory conclusions.

Tendinous tissue can also impose an influence upon the force-producing capabilities of the attached muscle. The stiffness of the tendon can influence the force-length relationship of its respective muscle, particularly at the extremes of the muscle range of motion (20), underpinned by a change in myofilament overlap in the sarcomeres of the muscle. When considering the effect of tendon stiffness on muscle and sarcomere length and consequent contractile force in different muscle-tendon units (MTUs), differences in tendon length and muscle length between MTUs should be considered, as for given tendon elongation will have to be shared by more in-series sarcomeres in longer muscles. One way of assessing differences in whole MTU stiffness between different MTUs is by comparing muscle fascicle: tendon length ratios between MTUs (21–23).

Significantly though, tendon stiffness is of course not the only influential factor when considering RTD; there are many characteristics of the muscle-tendon complex, which play a role (13,24). Muscle size, myosin heavy chain (MHC) composition, motor unit firing frequency, rate of EMG rise, and maximal strength are all known to influence RTD (4,10,24–28). Therefore, when investigating RTD, it seems imperative to consider all possible determinants including muscular, neural, and tendinous.

The purpose of this study was first, to establish whether patellar tendon stiffness undergoes any age-related changes. Second, we wanted to establish and quantify the effect of potential muscle and tendon factors affecting RTD.

## Methods

### Participants

We recruited 50 healthy, recreationally active individuals, 28 of which were younger males (YM, 23.9 years ± 1.1) and 22 older males (OM, 68.5 years ± 0.5). There were no differences in height (177 ± 1.3 vs 177 ± 1.3), mass (75.9 ± 3.2 kg vs 78.5 kg ± 2.3), or body mass index (BMI, 24.2 ± 0.8 vs 24.9 ± 0.6) between the YM and OM, respectively. All participants underwent a full medical screening prior to enrolment, whereby those with any musculoskeletal, metabolic, respiratory, neurological, or cardiovascular medical conditions were excluded from the study. None of the participants utilized in this study were deemed as frail, or had a history of falls. All participants provided informed written consent to this study, which was approved by the University of Nottingham Ethics Committee and complied with the Declaration of Helsinki.

### Assessment of Maximal Voluntary Contraction (MVC)

Isometric knee extensor torque during maximal voluntary contraction (MVC) was assessed via dynamometry whereby participants were seated upon a rigid tabletop dynamometer where the knee joint angle was set to 90°, with full extension equal to 0°. Participants were strapped tightly across the thigh, with their lower leg secured to a calibrated load cell via Velcro strapping. Force measures were acquired and passed through an analogue to digital converter (BioPac MP150, BIOPAC Systems Inc) sampling at 500 Hz and plotted using Acqknowledge software. Consequently the torque values were calculated by multiplying the obtained force values by the external moment arm. Subjects performed a total of three MVC attempts, each separated by 30 seconds. MVC was defined as the maximal torque value obtained over the three attempts.

### Electromyography (EMG)

EMG was acquired from the belly of both the vastus lateralis (VL) and the biceps femoris (BF) muscles of the right lower limb using surface EMG electrodes. The skin was thoroughly prepared through the removal of hair, light abrasion and cleaned with 0.1% w/w alcohol wipes. Raw EMG signals were sampled at 2.0kHz and digitized with an analogue to digital converter (BioPac MP150, BIOPAC Systems Inc) filtered and amplified. The root mean square (RMS) was calculated through an Acqknowledge software function (Acqknowledge 4.2.0, BIOPAC systems Inc), where the integral was calculated using a 30 ms time-window.

### Muscle Activation Capacity

Volitional muscle activation of the quadriceps was assessed using the interpolated twitch technique. Individuals produced a maximal knee extension at 90° knee joint angle, during which individuals received electrical stimulation using a stimulator device (DS7AH, dual-high voltage stimulator, Digitimer Ltd, Welwyn Garden City Hertfordshire, UK). The stimulus was applied via two pads, which were placed proximally and distally to the quadriceps. The stimulation consisted of two supra-maximal single pulses, one delivered when a plateau was reached during contraction, a second (potentiated twitch) was applied at rest 1-second postcontraction. The electrical current required for each individual’s supra-maximal stimulation was obtained prior to assessment. The supra-maximal current was acquired through monitoring force output during a succession of stimuli with increasing current. At the point whereby no additional force was observed for a further increase in current, the current was recorded and utilized during assessment. Muscle activation was consequently calculated via an equation previously used (29), which is listed below; whereby T_1_ is the evoked interpolated twitch torque and T_2_ is the resting control twitch torque.

$$Activation\;Capacity=\left(1-\left(\;\frac{T_{1}}{T_{2}}\;\right)\right)x\;100$$

The maximal first derivative (dF/dt) of the resting control twitch (T_2_) was also obtained as a marker of contractile speed.

### Rate of Torque Development (RTD)

For the acquisition of quadriceps femoris contractile RTD, participants were seated upon the rigid tabletop dynamometer used for acquisition of MVC. The VL of the right leg was prepared for EMG in the manner stated above. Similarly to the methods in which MVC was obtained, participants were strapped tightly across the thigh, with their lower leg secured to a calibrated load cell via Velcro strapping. Participants were instructed to produce a rapid and powerful contraction of the knee extensors, with emphasis upon speed. Subjects performed a trial repetition for familiarization, before completing three maximal isometric contractions, performed as fast as possible, each separated by 30 seconds of rest. Absolute RTD (aRTD) was defined as the change in force over a given period. We assessed RTD over the first 200 ms of the maximal isometric contraction, whereby the onset of contraction was deemed as the point at which torque had risen 4Nm above baseline. We also obtained torque values at 50 ms and 100 ms to generate an averaged curve of torque over the initial 200 ms of contraction. RTD values were consequently normalized (nRTD) for peak quadriceps anatomical CSA (ACSA). The time to peak EMG (TTPE) was calculated as the time taken from EMG onset to 90% of peak EMG amplitude during the maximal isometric contraction (4).

### Magnetic Resonance Imaging (MRI)

Muscle and patellar tendon dimensions cross sectional area (CSA) were measured along the full length utilizing a *3T MRI scanner*. Transversal scans were performed with participants lying supine, with the knee joint fixed at 180°. Peak quadriceps ACSA was manually measured using digital analysis software (OsiriX Lite 8, Pixmeo SARL). Tendon CSA was also manually measured; an average value was generated for CSA along the full length of the tendon to compensate for spatial variations in CSA.

### Tendon Biomechanical Properties

Measures of tendon biomechanical properties (tendon stiffness and young’s modulus) were obtained in vivo during voluntary isometric ramped contractions at 90° knee joint angle over a 5-second period. Patellar tendon length (L_0_) and hence elongation was obtained by sagittal-plane real-time B-mode ultrasonography with a 10 cm linear-array probe. *L*_0_ was defined as the distance between the apex of the patella and the first tuberosity of the tibia. Tendon elongation was calculated through utilizing a piece of automated pixel tracking software (Tracker v4.95, OpenPhysics), which was run in triplicate and the average pixel movement was used for further analysis. Moreover, to prevent an overestimation of tendon elongation due to translation of the tibia, a calibrated goniometer was attached to the lateral side of the tested knee. In order to calculate patellar tendon stiffness, true knee extensor torque was calculated as the sum of the measured knee extension torque and the antagonistic torque, estimated through the RMS of the EMG acquired from the Biceps Femoris (BF). Consequently the patellar tendon force was calculated by dividing the true knee extensor torque by the estimated patellar tendon moment arm (30). Force-elongation data were fitted with a second order polynomial curve, which allowed the assessment of patellar tendon stiffness, defined as the gradient of the force-elongation curve over the final 10% of maximal force (31). Tendon CSA (cm^2^) was assessed along the full resting length of the tendon in the axial plane through MRI as described above, with the average value being presented as patellar tendon CSA. Young’s modulus was calculated as tendon stiffness multiplied by the ratio of tendon length over tendon CSA.

### Muscle Architecture

VL fascicle length was assessed through the acquisition and analysis of images using B-mode ultrasonography (Mylab 70, Esaote Biomedica) with a 100 mm, 10–15 MHz, linear array probe. Images were obtained while the participant was at rest, and lying supine upon a bed, in the same manner as previous work (32). However, briefly, images were acquired from 50% of the VL length and midsagittal line of the muscle. The transducer was aligned to the fascicle plane allowing optimal capture of the fascicles (33). Data were collected and analyzed by the same operator to remove any inter-operator error. Digital analysis of the images was completed using imageJ (ImageJ 1.50i), whereby the fascicle length was directly measured wherever possible, or in the circumstance the fascicles extended beyond the visible field of view, linear extrapolation was applied (34).

### Statistical Analyses

All data are presented as mean ± SEM. Differences between the two groups (YM vs OM) were analyzed using unpaired two-tailed *t* tests (level of significance was set at *p* < .05). Linear regression analyses were assessed through Pearson’s correlation coefficient (level of significance was set at *p* < .05), similarly correlation matrices were also conducted in this fashion.

## Results

**Figure 1.**
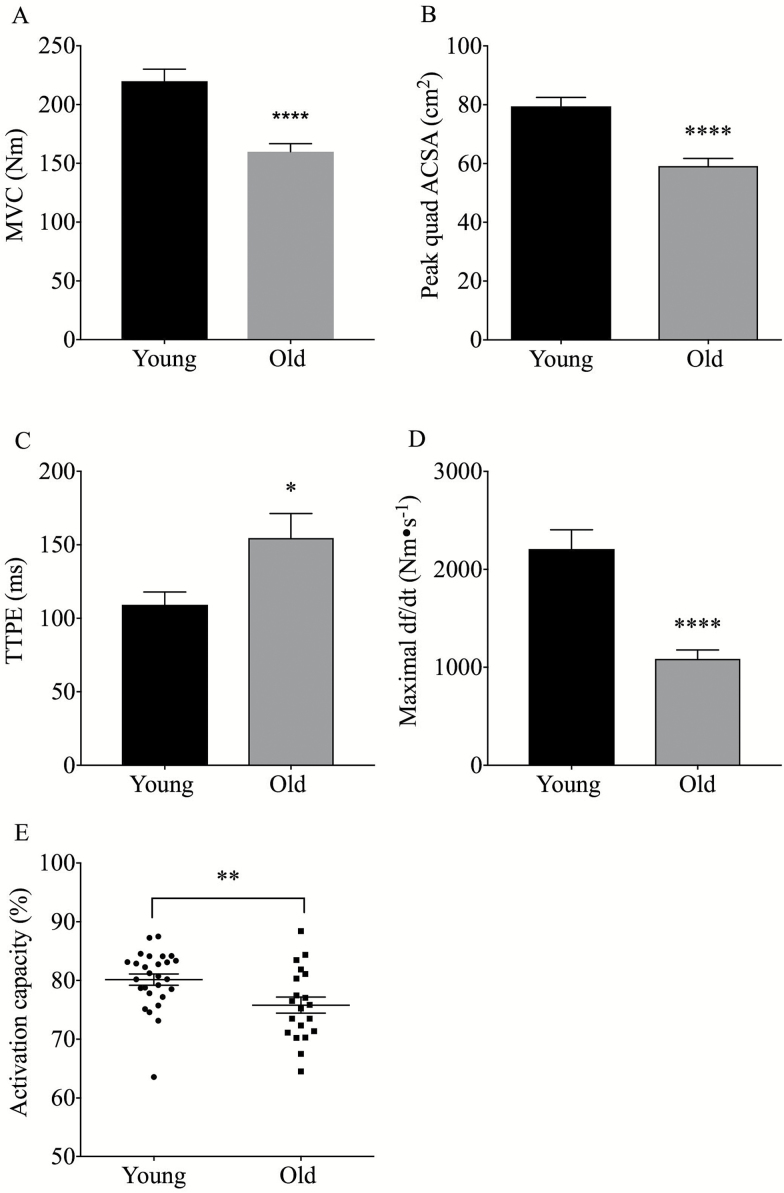
Age-related difference in MVC (**A**), Peak Quadriceps CSA (**B**), TTPE (**C**), Maximal twitch df/dt (**D**), and Activation capacity (**E**), *p* values denoted by *p < .05, **p < .01, ***p < .001, ****p < .0001. ACSA = Anatomical cross sectional area; MVC = Maximal voluntary contraction; TTPE = Time to peak EMG amplitude.

**Figure 2.**
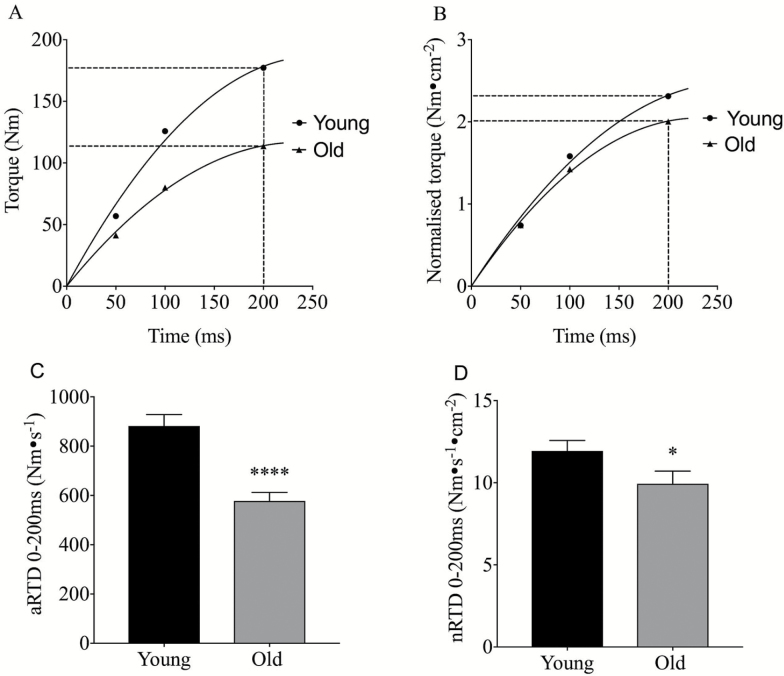
Graphs depict the average raw torque (**A**) and normalized torque (**B**) values obtained at 50, 100, and 200 ms during rapid isometric contraction. Age-related difference in aRTD (**C**) and nRTD (**D**), *p* values denoted by *p < .05, ****p < .0001. RTD = Rate of torque development.

OM demonstrated a lower isometric MVC (159 ± 6.8 Nm vs 220 ± 10 Nm, *p* < .0001) and a reduced quadriceps ACSA (59.1 ± 2.5 cm^2^ vs 79.4 ± 2.9 cm^2^, *p* < .0001) compared to YM (Figure 1). Quadriceps voluntary activation (AC) during maximal leg extension was significantly lower in OM than in YM (75.8 ± 1.5% vs 80.1 ± 0.9%, *p* < .01), and the muscle twitch maximal df/dt (1,086 Nm∙s^−1^ vs 2,209 Nm∙s^−1^, *p* < .0001) was also lower in OM (Figure 1). Also, TTPE was significantly slower in OM than in YM (154.6 ± 16.6 ms vs. 109.2 ± 8.6 ms, *p* < .05). Absolute RTD (aRTD), assessed over the first 200 ms of a maximal isometric contraction, was significantly reduced in OM (Figure 2) (577 ± 34 Nm∙s^−1^ vs 881 ± 46 Nm∙s^−1^, *p* < .0001). The age-related reduction in RTD was maintained after normalization of RTD to peak ACSA (nRTD) (Figure 2) (11.9 ± 0.64 Nm∙s^−1^∙cm^2^ vs 9.93 ± 0.77 Nm∙s^−1^∙cm^2^, *p* < .05).

**Figure 3.**
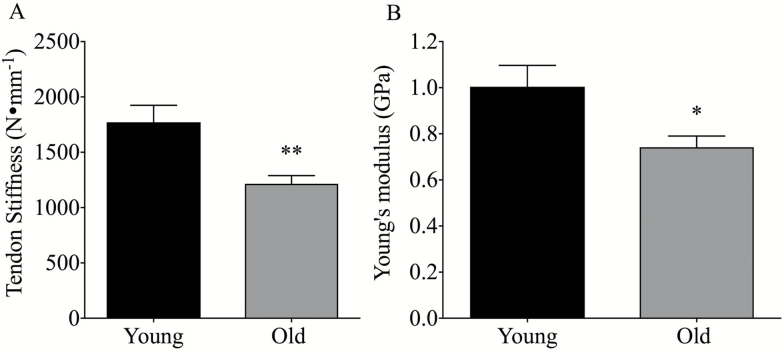
Age-related difference in patellar tendon stiffness (**A**) and Young’s Modulus (**B**), *p* values denoted by *p < .05, **p < .01.

Patellar tendon CSA (86.6.1 ± 2.6 mm^2^ vs 83.8 ± 2.7 mm^2^ for YM and OM, respectively, *p* > .05) and length (50.8 ± 0.9 mm vs 51.9 ± 0.8 mm for YM and OM, respectively, *p* > .05) were found to be unaffected by age. Patellar tendon stiffness assessed over the maximal 10% of the force elongation curve was found to be lower in OM than YM (Figure 3) (1,222 ± 78 N∙mm^−1^ vs 1,771 ± 154 N∙mm^−1^, *p* < .01). Young’s modulus was also lower in OM than YM (Figure 2) (0.74 ± 0.04 GPa vs 1.0 ± 0.09 GPa, *p* < .05). No differences in either fascicle length (YM: 85 ± 1.8 mm vs OM: 82.7 ± 3 mm, *p* > .05) or in resting fascicle: tendon length ratio (YM: 1.69 ± 0.04 vs OM: 1.58 ± 0.05, *p* > .05) were found between groups.

**Figure 4.**
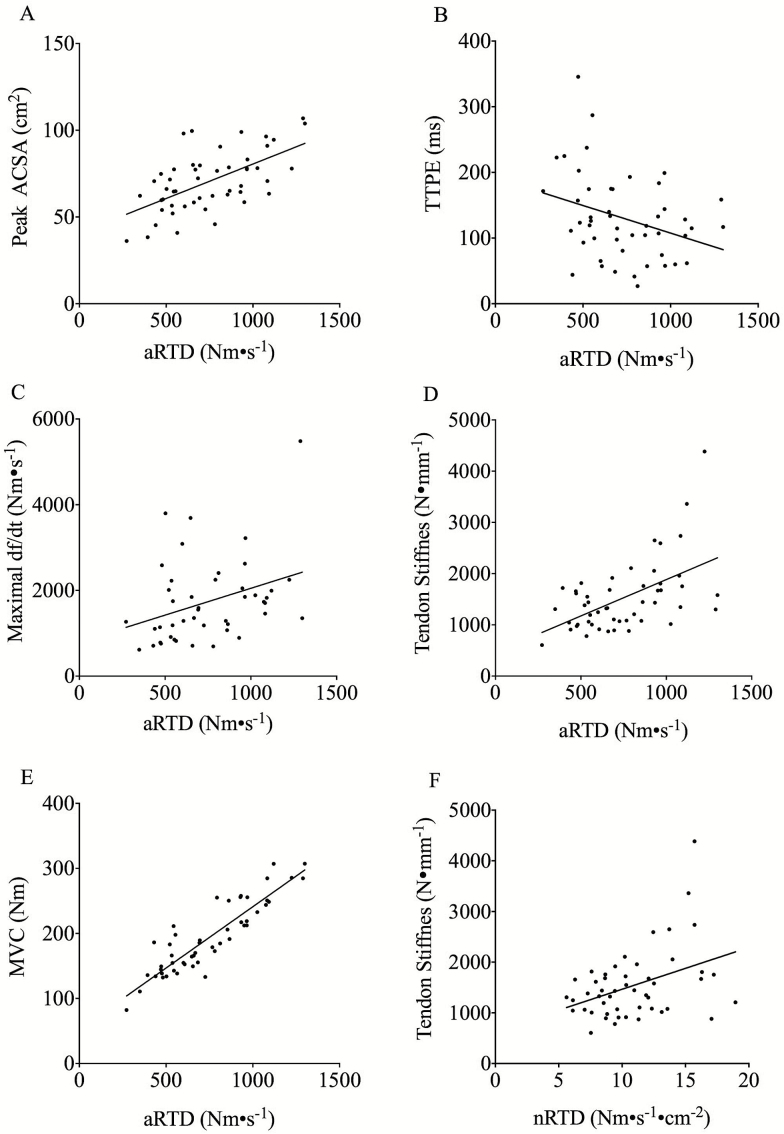
Graphs A–E demonstrate correlation between aRTD and peak quadriceps ACSA (**A**), TTPE (**B**), maximal twitch df/dt (**C**), tendon stiffness (**D**), and MVC Graph (**E**). The correlation between nRTD and Tendon stiffness is also shown (**F**). ACSA = Anatomical cross sectional area; MVC = Maximal voluntary contraction; RTD = Rate of torque development; TTPE = Time to peak EMG amplitude.

aRTD correlated (Figure 4 and Table 1) to MVC (*R*^2^ = .80, *p* < .0001), CSA (*R*^2^ = .36, *p* < .0001), tendon stiffness (*R*^2^ = .28, *p* < .0001), TTPE (*R*^2^ = .11, *p* < .05), and muscle twitch maximal df/dt (*R*^2^ = .11, *p* < .05). nRTD remained correlated with tendon stiffness (Figure 4 and Table 2) (*R*^2^ = .15, *p* < .01).

**Table 1.** Correlation Matrix for all Independent Variables With *r* Values Displayed

	Stiffness	MVC	CSA	TTPE	Max df/dt	FL
Stiffness	1	.605***; *p* < .001	.583***; *p* < .001	−.209; NS	.247; NS	.165; NS
MVC	.605***; *p* < .001	1	.703**; *p* < .001	−.122; NS	.347*; *p* < .05	.082; NS
CSA	.583***; *p* < .001	.703**; *p* < .001	1	−.122; NS	.587; *p* < .001	.018; NS
TTPE	−.209; NS	−.224; NS	−.122; NS	1	−.231; NS	.183; NS
Max df/dt	.247; NS	.347*; *p* < .05	.587***; *p* < .001	−.231; NS	1	−.029; NS
FL	.165; NS	.082; NS	.018; NS	.183; NS	−.029; NS	1

*Note*: CSA = Cross sectional area; FL = Fascicle Length; MVC = Maximal voluntary contraction; NS = Not significant; TTPE = Time to peak EMG amplitude. *p* values denoted by *p < .05, **p < .01, and ***p < .001.

**Table 2.** Correlation Matrix for aRTD and nRTD with *r* Values Displayed

	Stiffness	MVC	CSA	TTPE	Max df/dt	FL
aRTD	.534***; *p* < .001	.895***; *p* < .001	.600***; *p* < .001	−.320*; *p* < .05	.345*; *p* < .05	.144; NS
nRTD	.402**; *p* < .01	N/A	N/A	−.264; NS	.147; NS	.184; NS

*Note*: CSA = Cross sectional area; FL = Fascicle length; MVC = Maximal voluntary contraction; N/A, Not applicable; NS, Not significant; RTD = Rate of torque development; TTPE = Time to peak EMG amplitude. *p* values denoted by *p < .05, **p < .01, and ***p < .001

## Discussion

This study aimed to identify the origin of age-related differences in the rate of torque development in healthy older individuals. Our findings show that significant differences in the rate of torque development exist between young and older males both in terms of absolute RTD (aRTD) and RTD normalized (nRTD) for muscle size, thus accounting for age-related loss in muscle mass (sarcopenia). Notably, this study provides evidence that the observed decrease in RTD is accounted by (i) lower tendon stiffness, (ii) slower neuromuscular activation, and (iii) reduced ACSA and thus force output.

The results herein clearly demonstrate that at the maximal 10% of the force-elongation curve, the calculated patellar tendon stiffness was significantly lower in OM than YM. Moreover, Young’s modulus was also found to be significantly lower, an expected result, as no tendon dimensional differences were observed. Thus, these observations suggest that these biomechanical changes cannot be explained by differences in tendon dimensions per se but are likely due to alterations in material properties. The lack of differences in tendon dimensions between groups explains why MTU stiffness was not different or affected RTD. Tendon tissue material alterations may include changes in tendon hydration status, glycosamine concentration, changes in elastin content, changes in cross-linking, a change in overall collagen content or a reduction in collagen fibril diameter (17,35). However, the latter two may be excluded, since previous work has demonstrated that there is no change in tendon collagen content with ageing (36) and one would assume a reduction in tendon CSA would follow a reduction in fibril diameter, which was not detected herein. To thoroughly investigate the potential drivers behind a change in tendon stiffness, biochemical analysis of the tendon tissue would need to be performed.

Nonetheless, our finding that patellar tendon stiffness is lower in OM is contradictory to previous work (16,17); therefore, this is the first paper, to our knowledge, to demonstrate an age-related decline in patellar tendon stiffness. The two-aforementioned studies had smaller group sizes (*n* = 6) and large variations in stiffness measuring, possibly limiting the detection of age-related differences. Large variations in tendon stiffness are often observed as adaptation is dependent upon total exposure to strain and hence levels of activity. The level of daily activity in the elderly adults varies considerably and may therefore produce a wide range of values, supporting the need for larger cohorts. Unfortunately, our study did not employ any measure of habitual activity and therefore it is plausible that our elderly group, despite being healthy and fully independent, were somewhat less active than the young.

Notably, our data illustrate that the aRTD of OM is significantly reduced in comparison to YM. As aforementioned RTD is a multifactorial variable, however these data clearly show that maximal force production has the greatest influence (*R*^2^ = .52) when considering the absolute value of RTD. Inherently, there are additional neuromuscular factors which could contribute to the age-related decrease in force output including a reduction in quadriceps peak ACSA and quadriceps AC, both of which were lower in OM. The data also demonstrates an increase in the twitch CT in OM, which is known to reflect a shift in the distribution of muscle fibre MHC-II isoforms (26). Our findings therefore suggest that one of the key determinants of aRTD is the shortening velocity of the contractile component (12).

However, aRTD is also influenced by muscle force level of which one key factor is muscle size. Hence, if an individual is capable of larger force output, the value of RTD will inherently be higher simply because of allometric scaling. Accordingly, it is essential to normalize the absolute RTD value to muscle size to account for the influence of dimensional scaling on force production. Our findings show that following normalization, the age-related reduction in RTD is still present, indicating that RTD is not due simply to differences in muscle size. Significantly, after normalization to quadriceps ACSA, tendon stiffness still remains correlated to nRTD (*R*^2^ = .16). This is a key finding as it demonstrates that even when the main muscular and neuronal factors are accounted for, tendon stiffness may still influence the rate of force development. This has important implications for exercise interventions designed for the elderly adults. While it is very well known that muscle weakness in the elderly adults can be partially prevented through resistive exercise training, it is also known that tendon tissue adapts to exercise (19,37,38). Therefore, it is essential that these interventions should also target the tendon tissue to induce some restoration of tendon stiffness. Furthermore, the data presented herein was acquired from healthy/nonfrail individuals, while it is not clear to what extent these issues would present in the frail; it is possible these deficits may be greater, which would further emphasize the need to appropriately recondition both muscle and tendon in old age. While these alterations wouldn’t reduce the likelihood of tripping, the alterations would however, increase the individual’s ability to counteracting the trip and hence reduce either the incidence or severity of the fall.

Unfortunately, this study has the limitation of lacking force-velocity measures. Nonetheless, given the close correlation between contraction time and MHC-II (26), the observed decrease in maximal df/dt in the OM participants suggests that velocity of contraction would be lower in these subjects. Conversely, a decreased contraction time is reflective of an increased velocity of contraction (39,40). In addition, we also note that the data is cross-sectional in nature and inherently longitudinal data would be more apt in addressing the questions herein.

In conclusion, we have shown that a reduction in absolute and normalized knee extensor RTD with ageing is strongly influenced by an ageing-induced reduction in overall strength, but also in part due to a decrease in patellar tendon stiffness. Our study demonstrates evidence of a role of tendon stiffness and multiple neuromuscular factors in the decrease of RTD in older individuals.

## Funding

This project was supported by the Biotechnology and Biological Sciences Research Council (Grant number BB/K019104/1). PhD Studentship sponsored by Medical Research Council [Award Reference: 1397964].
